# ANALYSIS OF THE DEMAND AND INFLUENCING FACTORS FOR SMART HOME CARE AMONG COMMUNITY-DWELLING ELDERLY PEOPLE

**DOI:** 10.1093/geroni/igae098.4381

**Published:** 2024-12-31

**Authors:** HongFei Jia, YaPing Ding

**Affiliations:** Nanjing Medical University, Nanjing Medical University, Jiangsu, China (People’s Republic); Nanjing Medical University, Nanjing Medical University, Jiangsu, China (People’s Republic)

## Abstract

**Objective:**

To investigate the smart home care requirements for community-residing adults in their later years, analyze the factors influencing these needs, and establish a scientific basis for the development of targeted smart care solutions.

**Methods:**

Between December 2023 and February 2024, a convenience sampling method was employed to select 322 community-residing adults in their later years from Jiangsu Province as participants in this study. Assessment tools included a general information questionnaire, the Mini-Mental State Examination (MMSE), the Barthel Index for activities of daily living, and a custom scale to measure the demand for smart home care within this population. Data analysis involved independent samples t-tests, one-way ANOVA, and multiple linear regression to assess the smart home care needs and influencing factors among community-residing adults with disabilities in their later years.

**Results:**

The multiple linear regression analysis indicated that pre-retirement occupation, self-care ability, cognitive function, and the willingness to use smart care technologies in the future are significant factors influencing the demand for smart home care (all P-values < 0.05).

**Conclusion:**

The current demand for smart home care among community-residing adults in their later years exhibits diversity. To adequately meet their multifaceted smart care needs, it is essential to consider the main influencing factors such as their occupational background, level of self-care, and cognitive abilities.

